# Bacterial computing: a form of natural computing and its applications

**DOI:** 10.3389/fmicb.2014.00101

**Published:** 2014-03-25

**Authors:** Rafael Lahoz-Beltra, Jorge Navarro, Pedro C. Marijuán

**Affiliations:** ^1^Department of Applied Mathematics (Biomathematics), Faculty of Biological Sciences, Complutense University of MadridMadrid, Spain; ^2^Instituto Aragonés de Ciencias de la SaludZaragoza, Spain

**Keywords:** bacterial computing, genetic algorithms, bioinspired algorithms, natural computing, learning and evolution in artificial agents

## Abstract

The capability to establish adaptive relationships with the environment is an essential characteristic of living cells. Both bacterial computing and bacterial intelligence are two general traits manifested along adaptive behaviors that respond to surrounding environmental conditions. These two traits have generated a variety of theoretical and applied approaches. Since the different systems of bacterial signaling and the different ways of genetic change are better known and more carefully explored, the whole adaptive possibilities of bacteria may be studied under new angles. For instance, there appear instances of molecular “learning” along the mechanisms of evolution. More in concrete, and looking specifically at the time dimension, the bacterial mechanisms of learning and evolution appear as two different and related mechanisms for adaptation to the environment; in somatic time the former and in evolutionary time the latter. In the present chapter it will be reviewed the possible application of both kinds of mechanisms to prokaryotic molecular computing schemes as well as to the solution of real world problems.

## Introduction

Bacterial computing, as an applied field recently launched (Poet et al., 2010), as well as the theoretical approaches to prokaryotic or bacterial intelligence, are derived from the adaptive response of living cells to existing environmental conditions. From a practical standpoint, we could define bacterial computing as the possibility of using bacteria for solving problems that today are solved by computers. If a bacterium could perform the work of a computer, this would allow us to build millions of computers which be replicated every 30 min, and that they would be confined within a Petri dish. According to Amos (2011) natural computing paradigms inspired by biological processes (e.g., artificial neural networks, genetic algorithms, ant colony algorithms, etc.) have proved to be very effective. However, all these “forms of computing” occurs *in silico*, and therefore within a computer. At present and in agreement with Amos (2011), the challenge is the possibility to use biological substrates and biological processes to encode, store and manipulate information (Cordero et al., 2013). For instance, to build a simple computing device, using bacteria rather than silicon. Since the seminal work of (Adleman, 1994) the feasibility of using biological substrates for computing has been well-established: Levskaya et al. (2005) has shown that the living cell could be considered as a programmable computational device, Baumgardner et al. (2009) using DNA segments and Hin/hixC recombination system successfully programmed *E. coli* with a genetic circuit that enables bacteria to solve a classical problem in artificial intelligence, the Hamiltonian problem; or the theoretical model where bacteria are used to solve the “burnt pancake problem” (Heyer et al., 2010).

In a theoretical realm, bacterial computing could be an emergent phenomenon consequence of learning and evolution. Bacterial learning and evolution are but two different and related mechanisms for adaptation to the environment, in somatic time the former and in evolutionary time the latter (Di Paola et al., 2004). In this chapter we review the possible application of both mechanisms to prokaryotic molecular computing as well as to the solution of real world problems.

During recent years, some experiments have shown that bacteria can learn the ability to anticipate changes in their immediate environment. For instance, Tagkopoulos et al. (2008) found how *E. coli* colonies can develop the ability to associate higher temperatures with a lack of oxygen, and how bacteria have naturally “learned” to get ready for a serving of maltose after a lactose appetizer (Mitchell et al., 2009). According to Tagkopoulos et al. (2008), homeostasis explains microbial responses to environmental stimuli—by means of intracellular networks, microbes could exhibit predictive behavior in a fashion similar to metazoan nervous systems. Even more, bacteria are able to explore the environment within which they grow by utilizing the motility of their flagellar system (Lahoz-Beltra, 2007) and deploying a sophisticated “chemotactic” navigation system that samples the environmental conditions surrounding the cell and systematically guides *away* from the unfavorable conditions and *toward* the favorable ones.

In this chapter we review several theoretical studies, models, and simulations about bacterial forms of natural computing, gauging its potential application and impact. We review how proteins form molecular complexes and networks related to molecular signaling functions and bacterial information processing. Modeling proteins as McCulloch-Pitts neurons reviews a hardware model (Di Paola et al., 2004) demonstrating how proteins of the intervening signal transduction networks could be modeled as artificial neurons, simulating the dynamical aspects of the bacterial taxis. The model is based on the assumption that, in some important aspects, proteins (Di Paola et al., 2004) of the signaling system may be considered as McCulloch-Pitts artificial neurons (McCulloch and Pitts, 1943) that transfer and process information from the bacterium's membrane to the flagella motor.

Modeling proteins as networks of processing elements will review how these proteins are also involved in other bacterial signaling functions through complex molecular systems. Finally, Modeling metabolites as “metabolic hardware” suggests how similar “informational” properties of proteins, particularly enzymes, may organize cellular metabolism, by introducing in our study the concept of “metabolic hardware.”

Bacteria can also evolve some true learning behaviors to respond optimally to their environment (Bacterial evolution). At present, most methods in evolutionary computation are inspired in the fundamental principles of neo-Darwinism or population genetics theory, considering as main sources of variability chromosome crossover (or recombination) and mutation (Perales-Graván and Lahoz-Beltra, 2008). However, bacteria exhibit several other genetic mechanisms as sources of variability, i.e., mechanisms such as transformation, conjugation, and transduction. In Bacterial conjugation we discuss the applicability of conjugation, a genetic mechanism exhibited by bacterial populations, and we simulate the evolutionary process along this mechanism. The efficiency of bacterial conjugation (Perales-Graván and Lahoz-Beltra, 2008) is illustrated designing, by means of a genetic algorithm based on this very mechanism, an AM radio receiver. In Bacterial transduction we continue the study of bacterial evolution, in this case by modeling and simulating the whole bacterial transduction mechanism.

### Modeling proteins as McCulloch-Pitts neurons

**Figure 1:**
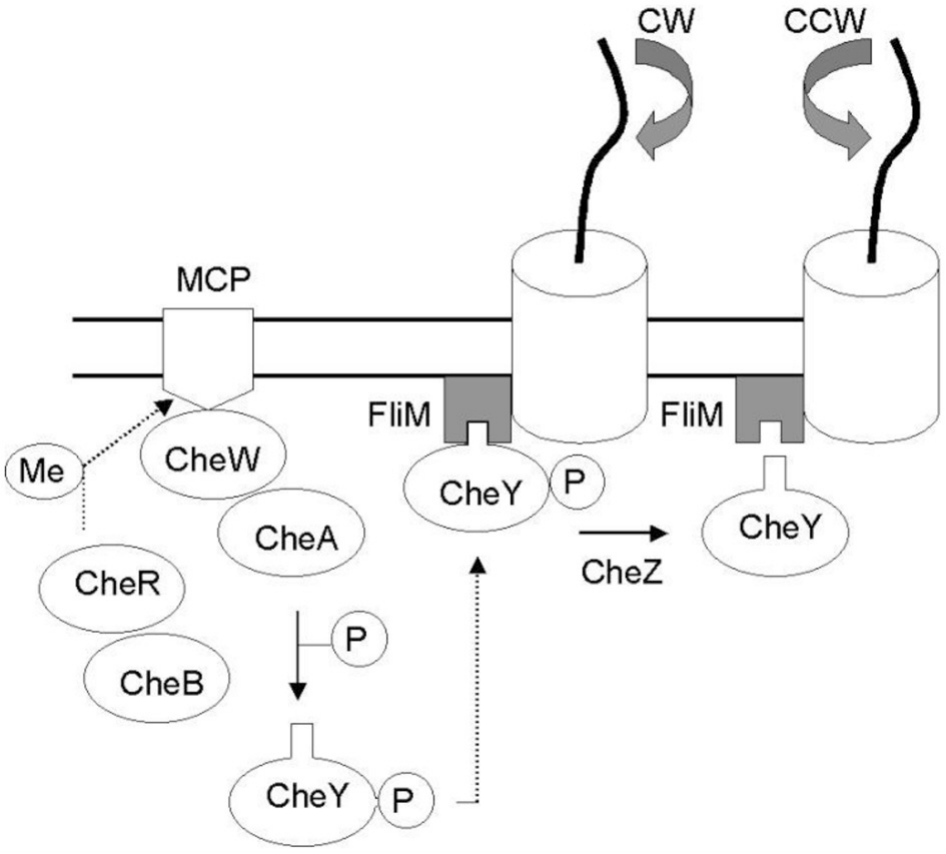
**Che protein network mediating bacterial taxis**. External factors are identified by membrane receptors (MCP) which reactivity depends on methylation levels controlled by CheB and CheR cytoplasmic proteins. In the network CheW is the bridge between the receptors and protein kinase CheA which can donates a phosphate group to CheY. The binding of CheY–P to the switch complex protein FliM induces a clockwise flagellar rotation (CW) whereas the dephosphorylation of CheY (via CheZ) restores counter-clockwise flagellar rotation (CCW) (Di Paola et al., 2004).

Adaptive behavior in bacteria depends on organized networks of proteins (Figure 1) governing molecular processes within the cellular system. Bacteria are able to explore the environment within which they develop by utilizing the motility of their flagellar system as well as a biochemical navigation system that samples the environmental conditions surrounding the cell (Di Paola et al., 2004). In this chapter we described how in some important aspects proteins can be considered as processing elements or McCulloch-Pitts artificial neurons that transfer and process information from the bacterium's membrane surface to the flagellar motor. The McCulloch-Pitts artificial neuron (Lahoz-Beltra, 2008) is a mathematical model (Di Paola et al., 2004) of a biological neuron. A neuron has a set of inputs *I*_1_, *I*_2_, …, *I*_*i*_, a set of weight values associated with each input line *W*_1_,*W*_2_,…,*W*_*i*_, one output *O*_*i*_ and a linear threshold function *f*(*net*_*i*_). Every time a neuron receives a set of input signals, performs the weighted sum (with the weights associated with each line) obtaining a *net*_*i*_ value, finally deciding its state or output with a threshold function:

(1) $$net_{i}=\sum_{i}w_{i}I_{i}$$

(2) $$O_{i}=\{\begin{array}{cc} 1 & net_{i}\geq \theta \\ 0 & net_{i}<\theta \end{array}$$

where θ is a threshold value.

The hardware model of the McCulloch-Pitts output artificial neuron assumes some plausible analogies between neurons and proteins. For instance, the connection between neurons, thus synapses as well as the neuron input and output would have their equivalent in the proteins on the concepts of bond strength, external factors (e.g., heat, ions, chemical substances, etc.) and conformational states related to catalysis and binding, respectively. In this analogy, it is also assumed that activation function represents cooperativity in proteins.

The hardware model of the proteins as a McCulloch-Pitts artificial neuron is based on a 741 inverting operational amplifier (Rietman, 1988). The operational amplifier simulates the bond strength, external factors, conformational states and cooperativity by means of a potentiometer (*R*_1_,*R*_2_,…,*R*_*n*_), voltage (*V*_1_,*V*_2_,…,*V*_*n*_), voltage (*V*_0_) and its saturation curve. In the circuit *R*_1_,*R*_2_,…,*R*_*n*_ are *n* variable resistors modeling the weights associated to connections among the *n* membrane receptors—playing the role of the input neurons—and the McCulloch-Pitts output neuron. Thus, variable resistors simulate the degree of influence of *n* inputs or external factors acting as *repellents* assuming one input per membrane receptor. The resistor *R*_*f*_ simulates the feedback in the output neuron being *V*_0_ the output voltage. Assuming the presence in the medium or environment of *n* inputs or external factors which presence is modeled as *V*_*i*_ (*i* = 1, …, *n*) input voltages with *R*_*i*_ (*i* = 1, …, *n*) weights or variable resistors. According this model (Di Paola et al., 2004) it follows that:

(3) $$V_{0}=-R_{f}\sum_{i\;=\;1}^{n}\frac{V_{i}}{R_{i}}$$

Once the input is applied, the value of the output is given by the LED diode state powered by the *V*_0_ voltage. Even though LED diode intensity changes according to the output voltage in our experiments we only considered two states of the LED diode. When LED diode state is switch-off state then it means an output equal to 0 simulating that CheY and FliM remain separated and by consequence the flagellar rotation is CCW, indicating the bacterium swimming behavior. Otherwise, when the LED diode is switched-on then it means an output equal to 1, simulating that CheY binds to FliM, and as a result the flagellar rotation is CW, indicating a bacterium tumbling behavior. The threshold between states simulates the critical level of phosphorylation in which CheY binds to FliM resulting in the transition from 0 (swimming behavior) to 1 (tumbling behavior).

**Figure 2:**
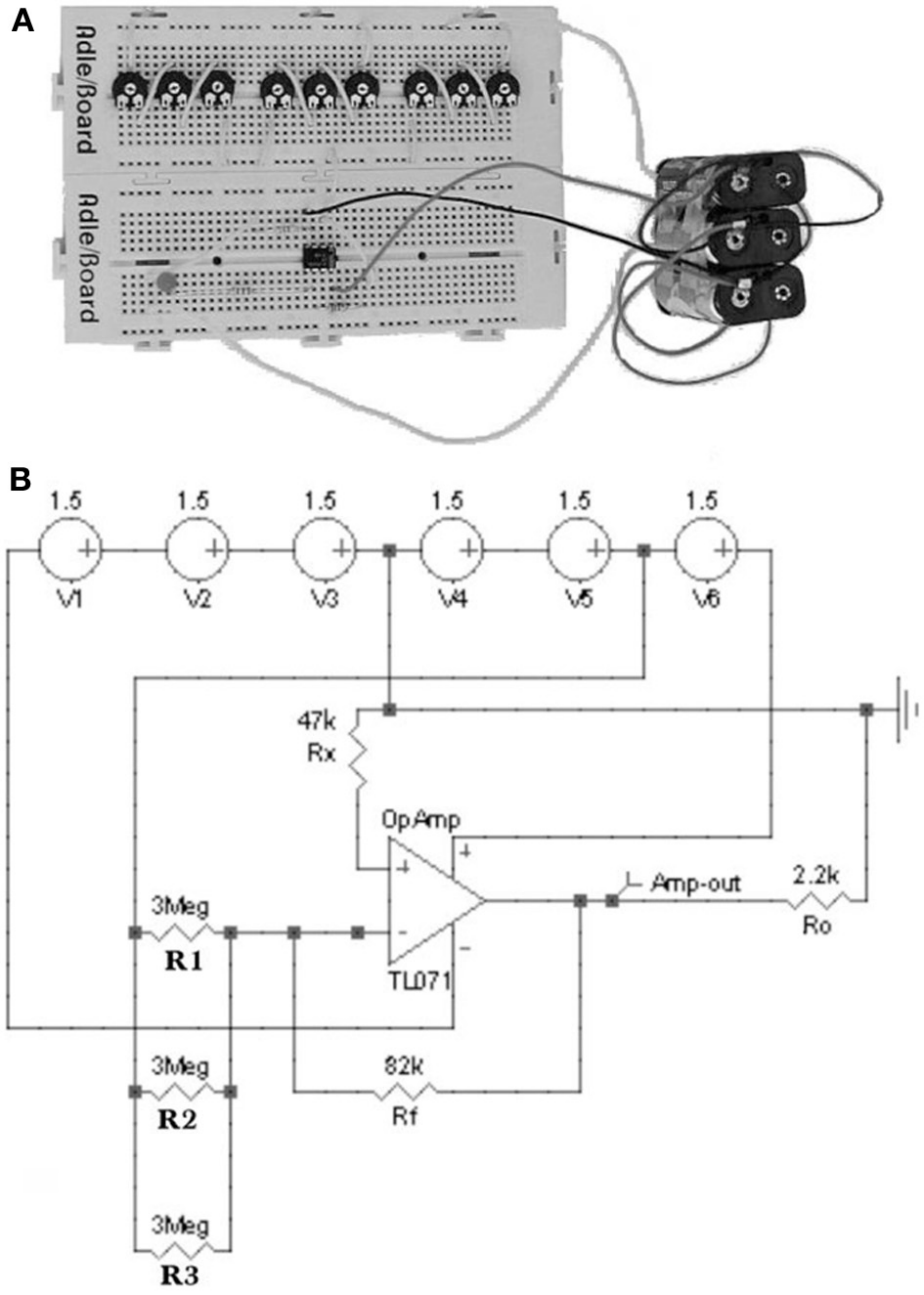
**(A)** Hardware and **(B)** software operational amplifier circuits with three repellent inputs. A voltage of +3V is applied to the potentiometers while the amplifier is powered with +9V (+4.5 and −4.5 V). In the hardware circuit **(A)** a LED diode connected to the amplifier output detects the intensity of the device signal. In the software circuit **(B)** a virtual voltage probe replaces to the LED diode (Di Paola et al., 2004).

In the practical implementation of the model, the amplification factor *A* of the operational amplifier:

(4) $$A=-R_{f}\sum_{i\;=\;1}^{n}\frac{1}{R_{i}}$$

simulates the catalytic effect of enzymes, e.g., the protein kinase CheA which donates a phosphate group to CheY resulting a phosphorylated CheY. As a consequence, if we establish a similarity between the *R*_*f*_ resistor and the Michaelis–Menten *K*_*m*_ constant then the resistor *R*_*f*_ would be simulating the affinity between the enzyme and substrate. Note that in our hardware model (Figure 2) the inhibitory connection that is usually included in the hardware implementation of artificial neural networks (Rietman, 1988) has been removed.

### Modeling proteins as networks of processing elements

**Figure 3:**
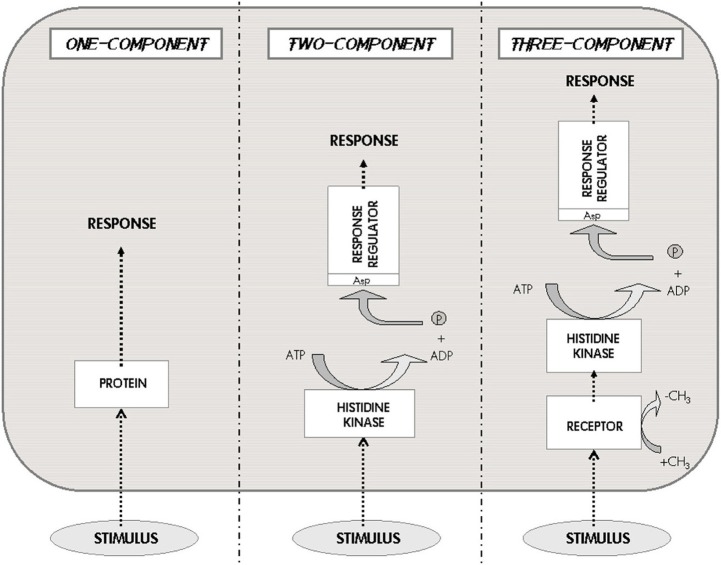
**The one-two-three Component Systems**. These three systems are the characteristic classes of signaling pathways developed by prokaryotes. The external stimulus is perceived either by an internal receptor–transducer (left), or by a transmembrane histidine kinase that connects with a response regulator (center), or by an independent receptor associated to the histidine kinase (right). This scheme represents the basic taxonomy of bacterial signaling; the three different options imply very different information processing capabilities and metabolic costs (Modified from Marijuán et al., 2010).

In general, the molecular systems involved in bacterial signaling (and in *M. tuberculosis*) are extremely diverse, ranging from very simple transcription regulators (single proteins comprising just two domains) to the multi-component, multi-pathway signaling cascades that regulate crucial stages of the cell cycle, such as sporulation, biofilm formation, dormancy, pathogenesis, etc. (Navarro and Marijuán, 2011). A basic taxonomy of bacterial signaling is shown in Figure 3. The first level of complexity corresponds to the simplest regulators, the “one-component systems.” Actually, most cellular proteins that participate in cellular adaptation to the changing environment, in a general sense, could be included as participating within this elementary category (Galperin et al., 2001). Following the complexity scale is the “two-component systems,” which include histidine protein-kinase receptors and an independent response regulator; they have been considered as the central signaling paradigm of the prokaryotic organisms, since a number of intercellular and interspecies communication processes are served by these systems. A further category (conceptual consistency) of “three-component systems” is applied to those two-component systems that incorporate an extra non-kinase receptor to activate the protein-kinase (Marijuán et al., 2010).

The Signaling/Transcriptional Regulatory Networks, like the M. tuberculosis network made for the authors of this paper (Sanz et al., 2011), may be used to analyze the ability of Mycobacterium to perceive the host signals in different tissues and cell types, as well as adaptive responses that bacteria organizes against them. In that sense, to adequately study on processes of latency and reactivation using these networks would be very important. These networks will be useful to provide an overview of multiple functional aspects of this bacterium and to suggest new experiments.

### Modeling metabolites as “metabolic hardware”

In this section we review the possibility of using metabolic networks as hardware in the study of the optimization of metabolic pathways as well as in the field of molecular and natural computing. We call to these bioinspired architectures as *metabolic hardware*. In particular, adopting as an example well known metabolic pathway of Krebs cycle, we introduce the methodology (Recio Rincon et al., 2013) to translate the molecular structure or topology of their metabolic intermediates to a binary matrix, showing how sugars and other glycolytic molecules could be modeled as binary matrices as well as LED dot matrices. In prokaryotic cells and bacteria which lack mitochondria, the Krebs cycle is performed in the cytosol.

**Figure 4:**
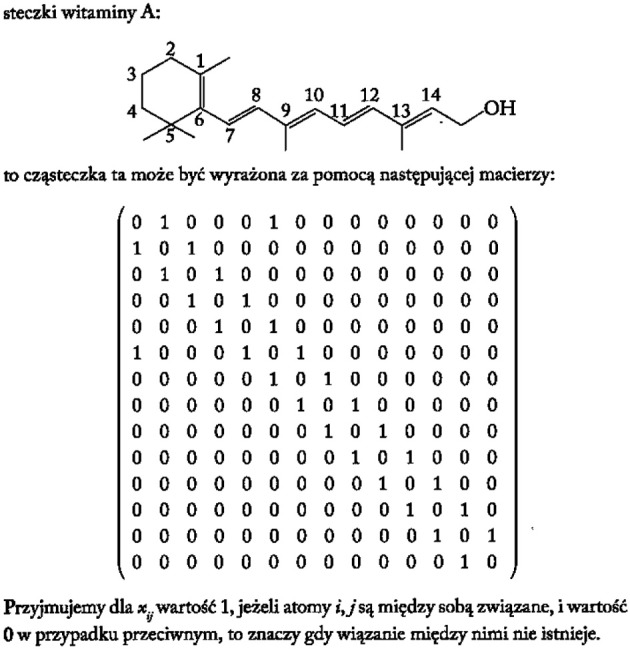
**The molecule of vitamin A or retinol represented as a binary matrix (Lahoz-Beltra, 2012a; Transl: Polish)**.

**Figure 5:**
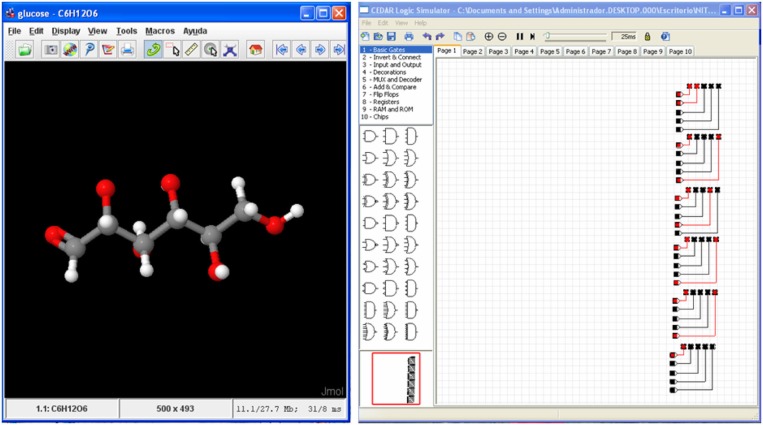
**Glucose molecule (Left) and its hardware version as a matrix of LEDs (Right) simulated with CEDAR Logic Simulator (Recio Rincon et al., 2013)**.

From a historical perspective one of the first procedures to translate the molecular topology to a matrix was introduced by Randic (1974), taking an element *a*_*ij*_ the value 1 when the vertices are adjacent or 0 otherwise. Figure 4 illustrates an example of this method for vitamin A or retinol (Lahoz-Beltra, 2012a). Our method assigns a 5-bit word to the functional groups of the molecule (Figure 5). For that purpose we define a table or Rosetta stone (Table 1) that includes the most frequent functional groups in metabolic intermediates, which were ordered by its redox potential (tendency of a functional group to acquire electrons).

**Table 1:**
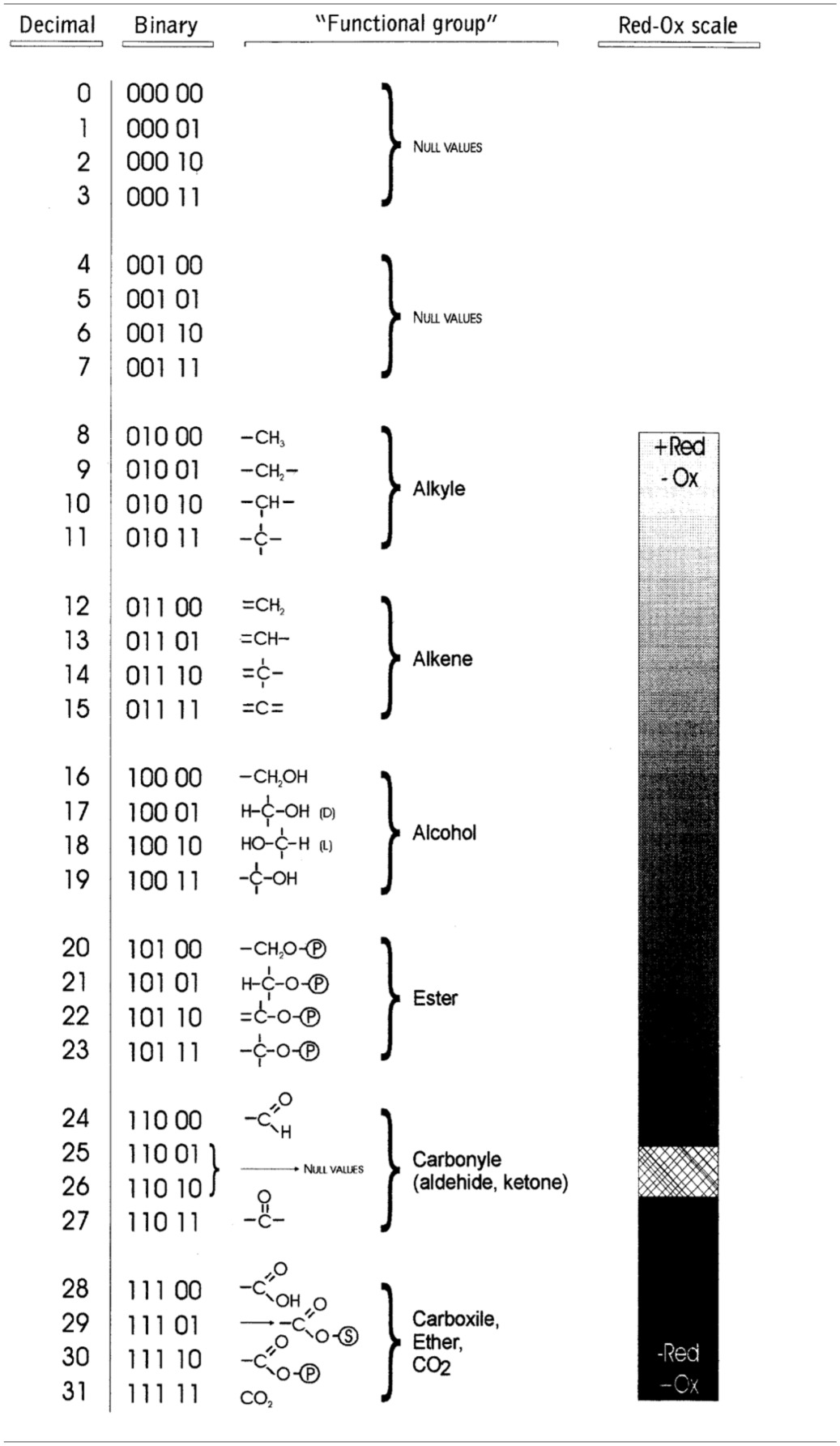
***Rosetta stone* for the hardware implementation of metabolic pathways**.

Let *S* and *P* be two binary matrices which represent respectively the substrate *S*_*m*_ and product *P*_*m*_ of a biochemical reaction catalyzed by an enzyme *E*_*m*_. Since in Krebs cycle all metabolites or metabolic intermediates are molecules with 4 or 5 carbon atoms, we defined (5) and (6) matrices respectively:

(5) $${\text{C}}_{4}=\left(\begin{array}{ccccc} a_{11} & a_{12} & a_{13} & a_{14} & a_{15}\\ a_{21} & a_{22} & a_{23} & a_{24} & a_{25}\\ a_{31} & a_{32} & a_{33} & a_{34} & a_{35}\\ a_{41} & a_{42} & a_{43} & a_{44} & a_{45} \end{array}\right)$$

(6) $${\text{C}}_{5}=\left(\begin{array}{ccccc} a_{11} & a_{12} & a_{13} & a_{14} & a_{15}\\ a_{21} & a_{22} & a_{23} & a_{24} & a_{25}\\ a_{31} & a_{32} & a_{33} & a_{34} & a_{35}\\ a_{41} & a_{42} & a_{43} & a_{44} & a_{45}\\ a_{51} & a_{52} & a_{53} & a_{54} & a_{55} \end{array}\right)$$

Note that given a value *i*, (*a*_*i*1_, *a*_*i*2_, …, *a*_*i*5_) is a row vector representing the functional group of the substrate *s*_*ij*_ or product *p*_*ij*_ molecules. Thus, each row in the matrices C_4_ and C_5_ represents a carbon atom in the molecule, having a total of 32 possible binary vectors from 00000 to 11111 (Table 1). Using as a criterion the redox potential vectors were classified from its most reduced (addition of hydrogen or the removal of oxygen) form or alkyl group to the most oxidized (addition of oxygen or the removal of hydrogen) or CO_2_. However, since the metabolites of Krebs cycle are the result of assembling functional groups among a total of 22 combinations of carbon, then 10 binary vectors are without chemical meaning. In order to perform future simulation experiments, molecules of CO_2_ and acetyl-CoA were represented as a row vector (7) and 2 × 5 matrix (8) shown below:

(7) $${\text{CO}}_{2}=\left(\begin{array}{ccccc} 1 & 1 & 1 & 1 & 1 \end{array}\right),$$

(8) $$\text{acetyl-CoA}=\left(\begin{array}{ccccc} 1 & 1 & 1 & 0 & 1\\ 0 & 1 & 0 & 0 & 0 \end{array}\right)$$

Using this method the Krebs cycle was modeled as follows (Recio Rincon et al., 2013):

$$\begin{array}{l} \begin{array}{c} \begin{array}{c} \left(\begin{array}{ccccc} 1 & 1 & 1 & 0 & 0\\ 0 & 1 & 0 & 0 & 1\\ 1 & 0 & 0 & 1 & 1_{\to }\\ 0 & 1 & 0 & 0 & 1\\ 1 & 1 & 1 & 0 & 0 \end{array}\right)\\ \leftarrow \left(\begin{array}{c} \begin{array}{ccccc} 1 & 1 & 1 & 0 & 0 \end{array} \end{array}\right) \end{array} \end{array}\to \begin{array}{c} \begin{array}{c} \left(\begin{array}{ccccc} 1 & 1 & 1 & 0 & 0\\ 0 & 1 & 0 & 0 & 1\\ 0 & 1 & 0 & 1 & 0_{\to }\\ 1 & 0 & 0 & 1 & 0\\ 1 & 1 & 1 & 0 & 0 \end{array}\right)\\ \leftarrow \left(\begin{array}{ccccc} \begin{array}{c} 1 \end{array} & 1 & 1 & 0 & 0 \end{array}\right) \end{array} \end{array}\begin{array}{c} \to \left(\begin{array}{ccccc} 1 & 1 & 1 & 0 & 0\\ 0 & 1 & 0 & 0 & 1\\ 0 & 1 & 0 & 0 & 1\\ 1 & 1 & 0 & 1 & 1\\ 1 & 1 & 1 & 0 & 0 \end{array}\right) \end{array}\begin{array}{c} \to \left(\begin{array}{ccccc} 1 & 1 & 1 & 0 & 0\\ 0 & 1 & 0 & 0 & 1\\ 0 & 1 & 0 & 0 & 1\\ 1 & 1 & 1 & 0 & 1 \end{array}\right)\\ \;↓ \end{array} \end{array}\begin{array}{c} \;\left(\begin{array}{ccccc} 1 & 1 & 1 & 0 & 0\\ 1 & 1 & 0 & 1 & 1\\ 0 & 1 & 0 & 0 & 1\\ 1 & 1 & 1 & 0 & 0 \end{array}\right)\leftarrow \left(\begin{array}{ccccc} 1 & 1 & 1 & 0 & 0\\ 0 & 1 & 0 & 0 & 1\\ 1 & 0 & 0 & 1 & 0\\ 1 & 1 & 1 & 0 & 0 \end{array}\right)\leftarrow \left(\begin{array}{ccccc} 1 & 1 & 1 & 0 & 0\\ 0 & 1 & 1 & 0 & 1\\ 0 & 1 & 1 & 0 & 1\\ 1 & 1 & 1 & 0 & 0 \end{array}\right)\leftarrow \left(\begin{array}{ccccc} 1 & 1 & 1 & 0 & 0\\ 0 & 1 & 0 & 0 & 1\\ 0 & 1 & 0 & 0 & 1\\ 1 & 1 & 1 & 0 & 0 \end{array}\right) \end{array}$$

where each matrix stands for one of the following metabolites: Citrate→ Iso-citrate → α-Ketoglutarate→ Succinyl-CoA → Succinate → Fumarate → Malate → Oxalacetate.

## Bacterial evolution

At present, all methods in Evolutionary Computation (genetic algorithms, evolutive algorithms, genetic programming, etc.) are bioinspired by the fundamental principles of neo-Darwinism (Lahoz-Beltra, 2008), and by a vertical gene transfer; that is to say, by a mechanism in which an organism receives genetic material from the ancestor from which it evolved (Perales-Graván et al., 2009). Indeed, most thinking in Evolutionary Computation focuses upon vertical gene transfer as well as upon crossover and/or mutation operations.

Bacteria are microscopic organisms whose single cells reproduce by means of a process of binary fission or of asexual reproduction, bearing a resemblance to John von Neumann's universal constructor (Von Neumann, 1966). Thus, a bacterial population (or colony) evolves according to an evolutive algorithm similar to Dawkin's biomorphs (Dawkins, 1986), the cumulative selection of mutations powering their evolution. Bacteria, however, exhibit significant phenomena of genetic transfer and crossover between cells. This kind of mechanism belongs to a particular kind of genetic transfer known as horizontal gene transfer. Horizontal, lateral or cross-population gene transfer is any process in which an organism, i.e., a donor bacterium, transfers a genetic segment to another one, a recipient bacterium, which is not its offspring. In the realm of biology, whereas the scope of vertical gene transfer is the population, in horizontal gene transfer the scope is the biosphere. This particular mode of parasexuality between “relative bacteria” includes three genetic mechanisms: conjugation, transduction and transformation. Furthermore, microorganisms are very interesting individuals because they also exhibit “social interactions.” We found (Lahoz-Beltra et al., 2009) how the inclusion of the “social life of microorganisms” into the genetic algorithm cycle, significantly improves the algorithm's performance.

In this section we explore the possibility of using for practical purposes some of the observed genetic transfer mechanisms in bacteria.

### Bacterial conjugation

This section describes a biologically inspired conjugation operator simulating a bacterial conjugation. Its usefulness is illustrated in a set of computer simulation experiments where including such operator into a genetic algorithm we were able to design an AM radio receiver (Perales-Graván and Lahoz-Beltra, 2008). The attributes optimized by this algorithm include the main features of the electronic components of an AM radio circuit, as well as those of the radio enclosure designed to house the radio circuit (Perales-Graván and Lahoz-Beltra, 2008).

**Figure 6:**
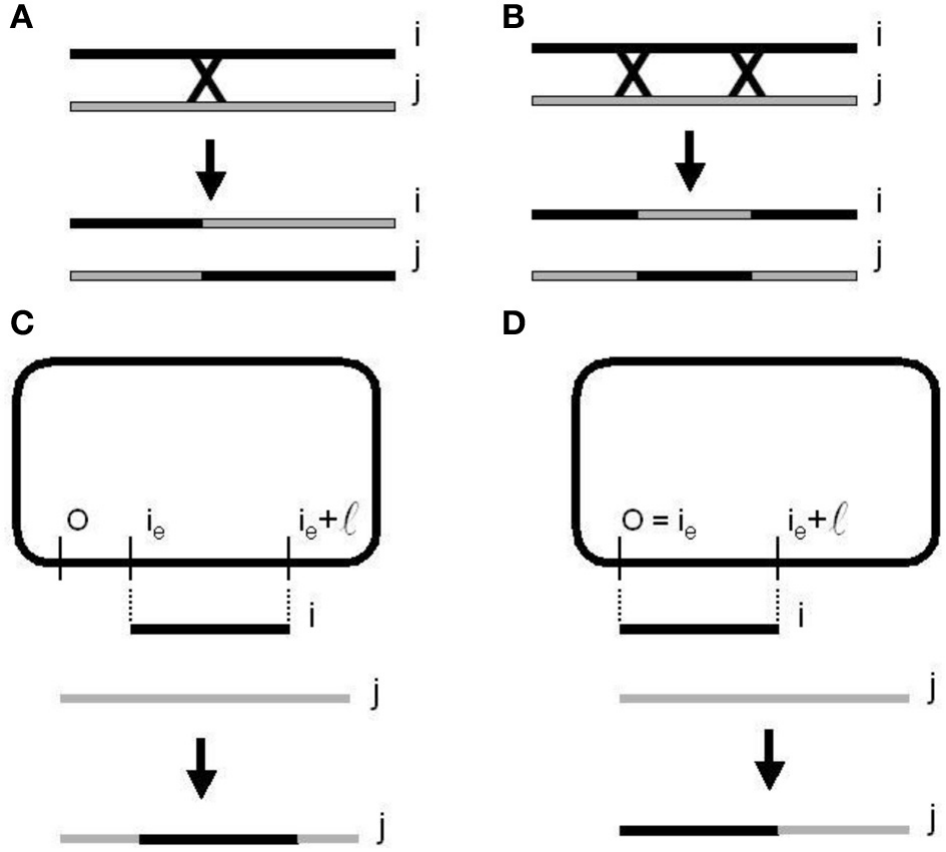
**Homologous recombination or cross over mechanisms. (A)** One-point re-combination. **(B)** Two-points recombination. Bacterial conjugation and recombination. **(C)** With a random point (CORP). **(D)** With a fixed point (COFP) on the donor chromosome (Perales-Graván and Lahoz-Beltra, 2008).

A bacterial genetic algorithm is an evolutionary strategy based on bacterial conjugation and mutation. Starting with a random population of circular chromosomes reproduction, conjugation and mutation were simulated, obtaining new generations of equal size. The current bacterial algorithm uses homologous recombination after conjugation, a population size and a conjugation parameter, as well as a conjugation (or recombination) and mutation probabilities (Perales-Graván and Lahoz-Beltra, 2008). Note that in the biological realm as well as in the simulations, the term population could be substituted by strain or colony and the linear chromosomes of a genetic algorithm are replaced by circular chromosomes. Our operator which includes the recombination between bacterial chromosomes assumes that donor bacterium is always Hfr. Two different conjugation operator versions (Perales-Graván and Lahoz-Beltra, 2008) have been defined (Figure 6). In both definitions since transfer of the donor bacterial chromosome is almost never complete, then the length of the strand transferred to the recipient cell has been simulated applying Monte Carlo's method and assuming DNA lengths exponentially distributed:

(9) $$l=-\frac{1}{\alpha }\ln(U)$$

being *U* a random number and α the conjugation parameter. The conjugation parameter summarizes all the relevant factors affecting the length *l* value. One of the most relevant factors affecting value is the temperature promoting the agitation of the bacteria, disrupting conjugation before the entire chromosome can be transferred (Perales-Graván and Lahoz-Beltra, 2008).

In bacteria, crossing over involves the aligning of the donor chromosome segment with its homologous segment on the recipient bacterial chromosome. Next, a break occurs at a point origin and an end point of the recipient chromosome, removing and replacing the segment with corresponding homologous genes from the segment of the donor chromosome (Perales-Graván and Lahoz-Beltra, 2008). The described steps are repeated several times, thus a number of times equal to the bacteria population size. The efficiency of the bacterial conjugation operator has been illustrated designing an AM radio receiver with a genetic algorithm based on this operator (Perales-Graván and Lahoz-Beltra, 2008).

**Figure 7:**
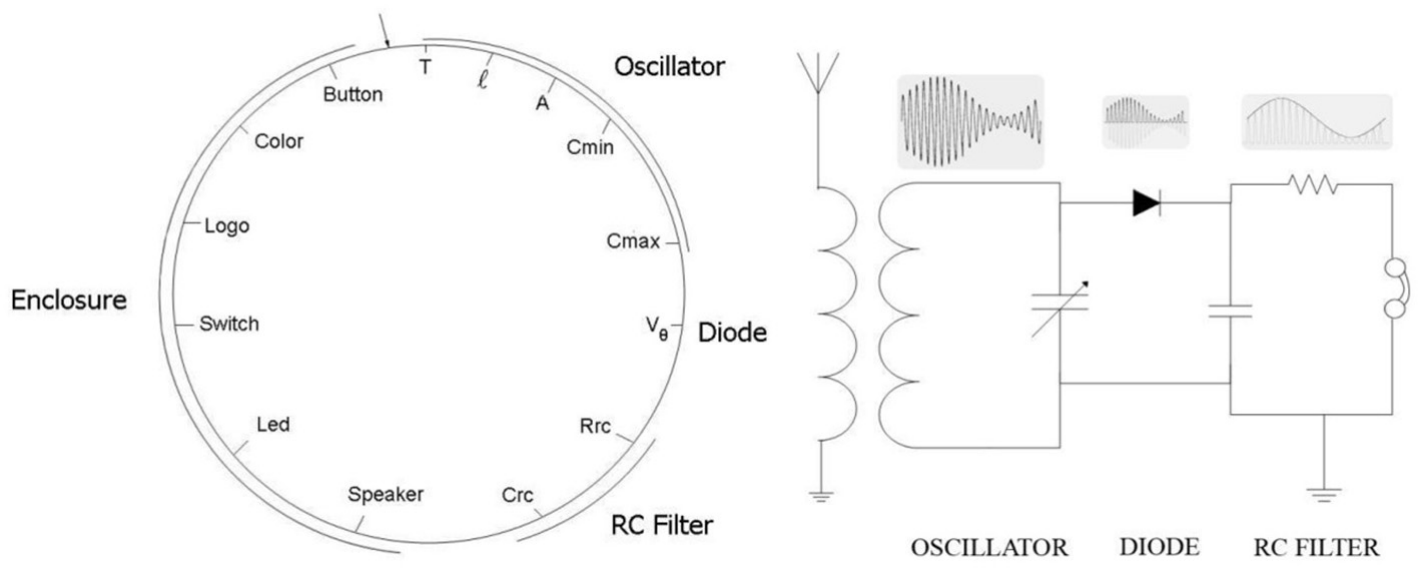
**Bacterial chromosome (left) with 14 genes codifying for the main characteristics of an AM radio receiver (right) (Perales-Graván and Lahoz-Beltra, 2008)**.

**Figure 8:**
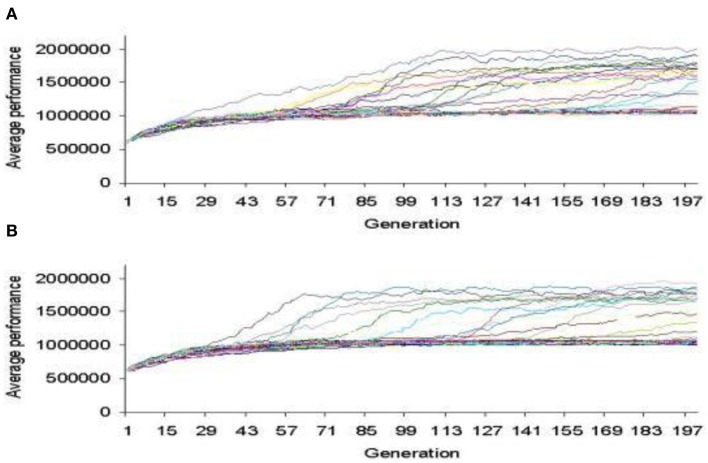
**Performance graph where bacterial colonies evolved searching for an optimized AM radio receiver. (A)** CORP. **(B)** COFP (Perales-Graván and Lahoz-Beltra, 2008).

In Figure 7, we show the bacterial chromosome coding for the main features of the radio receiver and in Figure 8 a representative performance graph (average fitness per generation) of the experiments where simulated bacterial colonies evolved searching for the optimized circuit and enclosure.

### Bacterial transduction

In Nature, microorganisms such as bacteria and viruses share a long and common evolutionary relationship (Perales-Graván et al., 2009). This relationship is mainly promoted by bacteriophages (or phages), a kind of virus that multiplies inside bacteria by making use of the bacterial biosynthetic machinery. Some bacteriophages are capable of moving bacterial DNA (the “bacterial chromosome”) from one bacterium to another. This process is known as transduction. When bacteriophages infect a bacterial cell, their normal mode of reproduction makes use of the bacterium's replication machinery, making numerous copies of its own viral genetic material (i.e., DNA or RNA). The nucleic acid copies (or chromosome segments) are then promptly packaged into newly synthesized copies of bacteriophage virions. Generalized transduction occurs when “any part” of the bacterial chromosome (rather than viral DNA) hitchhikes into the virus (i.e., T4 phages in *E. coli* bacterium). However, when only “specific genes” or certain special “segments” of the bacterial chromosome can be transduced, such a mistake is known as specialized transduction (i.e., λ phages in *E. coli* bacterium).

In transduction, transference of chromosome segments between bacterial populations or colonies is very different from migration (the occasional exchange of individuals). Migration and transduction could bear a resemblance, but only when transduction involves the complete chromosome transference between bacterial populations. Furthermore, this kind of transference is a highly unlikely event in bacteria, transduction of chromosome segments taking place in these microorganisms.

**Figure 9:**
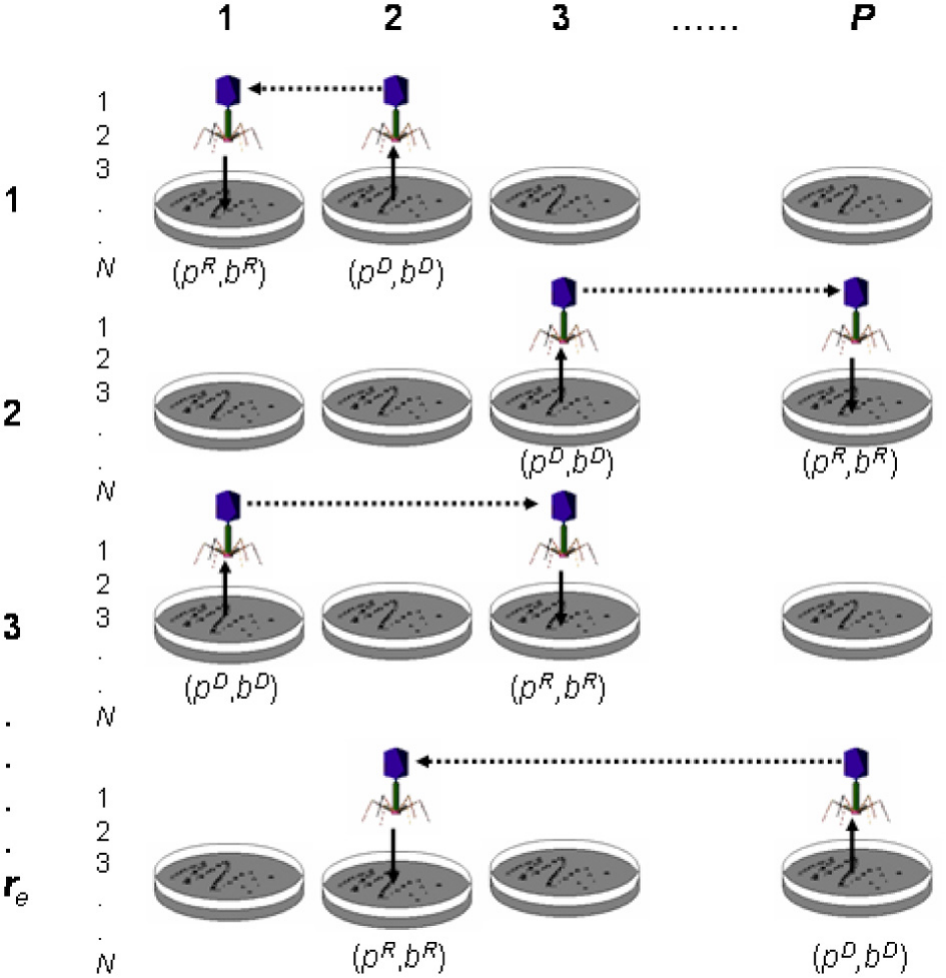
**Transduction experiment**. The figure shows transduction from donor Petri dish (*p*^*D*^) and bacterium (*b*^*D*^) to recipient Petri dish (*p*^*R*^) and bacterium (*b*^*R*^). In the figure, *P* is the total number of Petri dishes (or sub-populations), *N* is the number of bacteria (or population size) per Petri dish and *r*_*e*_ the number of experimental replicates.

In this section, we model and simulate the two kinds of transduction operations (Figure 9) examining the possible role and usefulness of this genetic mechanism in genetic algorithms (Perales-Graván et al., 2013). In a previous section (Perales-Graván and Lahoz-Beltra, 2008), we introduced a bacterial conjugation operator showing its utility by designing an AM radio receiver. Conjugation is one of the key genetic mechanisms of horizontal gene transfer between bacteria. In the present section, we refer to a genetic algorithm including transduction as PETRI (Promoting Evolution Through Reiterated Infection). We investigated the transfer of genes and chromosomes among sub-populations with a simulated “bacteriophage.” In the model we consider a structured population divided among several sub-populations or “bacterial colonies,” bearing a resemblance with coarse-grain distributed genetic algorithms. Each sub-population is represented as a Petri dish (a glass or plastic cylindrical dish used to culture microorganisms). It should be noted, however, that even when we divide a population into sub-populations, the proposed algorithm is sequential. Thus, the algorithm is not a distributed one, since we used a mono-processor computer and the algorithm was not parallelized. Moreover, the migration mechanism is synchronous, as gene and chromosome transferences were both between sub-populations and during the same generation. Therefore, our approach could be related with those models of Cellular Genetic Algorithms (cGA) adopted also for mono-processor machines (Alba and Dorronsoro, 2008), with no relation to parallelism at all. In our model, we assumed that bacteria are capable of displaying crossover through conjugation, instead of performing one-point or two-point recombination. Moreover, we assume that no vertical gene transfer mechanism is present in bacterial populations.

With the aim of studying the performance of the transduction operator, we used different optimization problems. Experiments conducted in the presence of transduction were compared with control experiments, performed in the absence of transduction. Similarly, we compared the transduction performance under the three types of crossover: conjugation, one-point or two-point recombination. We are interested in the study of genetic algorithms based on horizontal gene transfer mechanisms, mainly conjugation and transduction operations. It is important to note that even when conjugation and transduction are both horizontal gene transfer mechanisms, there are some relevant differences between both. In the first place, whereas conjugation involves two bacteria from the same population, the bacteria involved in transduction can belong to different populations. As a consequence, conjugation is a genetic mechanism of horizontal gene transfer within a population, whereas transduction is a genetic mechanism of horizontal gene transfer between populations. Secondly, in conjugation, the length of the transferred genetic segment is variable, whereas in transduction, the transferred segment length is always constant.

Let *b* be a chromosome (i.e., bacterium; 1, …, *j*, …, *N*) and *p* a sub-population (i.e., Petri dish; 1,…, *i*,…, *P*); then a transduction operation (Figure 9) is defined as follows: transduction is the transfer of genetic material from a Petri dish and bacterium donors (*p*^*D*^, *b*^*D*^) to a Petri dish and bacterium recipients (*p*^*R*^, *b*^*R*^). When the transference involves a chromosome segment, the result is a recombinant chromosome in the recipient Petri dish *p*^*R*^. However, the transference of a complete chromosome results in the substitution of one chromosome of the recipient Petri dish *p*^*R*^ with the transferred one. It is important to note that “bacterium” and “Petri dish” terms are used throughout the paper as “chromosome” and “sub-population” synonyms, respectively. Transduction requires the selection of the Petri dish and bacterium donors (*p*^*D*^, *b*^*D*^), as well as the Petri dish and bacterium recipients (*p*^*R*^, *b*^*R*^). In the reference (Perales-Graván et al., 2013) we describe how transduction was conducted.

The current PETRI algorithm (Figure 9B) uses a population size of *N*, performing *r*_*e*_ replicates, with *P* being the total number of Petri dishes or sub-populations. Thus, we performed a number of *r*_*e*_. *P* trials of each simulation experiment. The algorithm cycles through epochs, searching for an optimum solution until a maximum of *G* generations is reached. Once (*p*^*D*^, *b*^*D*^) and (*p*^*R*^, *b*^*R*^) are selected, only one “bacteriophage” is assumed to participate during each transduction event. The PETRI algorithm is summarized in the following pseudocode description:


/^*^ PETRI: Genetic Algorithm with Transduction ^*^/

1. t:=0;

2. Initialization: Generate P Petri dishes (or sub-populations)
    with N random bacteria (or chromosomes).

3. WHILE not stop condition DO

 /^*^ Genetic Algorithm ^*^/

   (3.1) FOR each P Petri dish DO

   Evaluation of chromosomes

    Selection

    Conjugation or Crossover (one-point, two-point)

    Mutation

   (3.2) END FOR

   /^*^ End of Genetic Algorithm ^*^/

4. Transduction: (pD, bD) (pR, bR)

5. t:=t+1;

6. END WHILE;

/^*^ End of PETRI ^*^/


We studied the performance of the simulated transduction by considering three optimization problems that are described in sufficient detail in Perales-Graván et al. (2013). The first problem uses a benchmark function, the second one is the 0/1 knapsack problem, and finally we illustrated the usefulness of transduction in the problem of designing an AM radio receiver (Perales-Graván and Lahoz-Beltra, 2008).

## Conclusions

In this chapter we have reviewed some of the models, simulations and theories that we have been working in recent years. The main conclusion of our work is that the bacterial cell can be seen as a form of natural computing (Lahoz-Beltra, 2012b), to which we have referred to in this chapter as “bacterial computing.” In bacteria computing capability emerges from two related processes: learning and evolution, being illustrated in this chapter several examples of hardware inspired in these processes. The possible impact of bacterial computing is not only to show how evolvable hardware can be used as a modeling framework in the simulation of learning and evolution in bacteria, also it promotes how electronic circuits could be designed based on “bacterial algorithms.”

In future, there will be wide range of applications of bacterial computing. For instance, recently Ran et al. (2012) designed a device made of DNA inserted into bacterial cell that works like a diagnostic computer. This molecular device works like a NOR logical gate being programmed to check for the presence of two transcription factors in such a way that responds by creating a protein that emits a green visible light—a sign of a positive diagnosis. Also, a few year ago a new technique has been developed to save data in bacteria. According to Yachie et al. (2007) up to 100 bits of data can be saved in each microorganism. Scientists successfully encoded and saved the phrase “*e* = *mc*^2^ 1905” the DNA of *Bacillus subtilis*. However, at present bacterial computing is a branch of synthetic biology. At present, bacterial computing depends of synthetic biology and the latter is still in its early stages. In order to design and build genuine “bacterial computers” we will need a better understanding of the operation of complex biological systems, i.e., bacteria.

### Conflict of interest statement

The authors declare that the research was conducted in the absence of any commercial or financial relationships that could be construed as a potential conflict of interest.
